# Advancements in bioengineered and autologous skin grafting techniques for skin reconstruction: a comprehensive review

**DOI:** 10.3389/fbioe.2024.1461328

**Published:** 2025-01-07

**Authors:** Jillian Dean, Cosima Hoch, Barbara Wollenberg, Justin Navidzadeh, Bhagvat Maheta, Anisha Mandava, Samuel Knoedler, Khalil Sherwani, Helena Baecher, Alina Schmitz, Michael Alfertshofer, Max Heiland, Kilian Kreutzer, Steffen Koerdt, Leonard Knoedler

**Affiliations:** ^1^ School of Medicine, University of Pittsburgh, Pittsburgh, PA, United States; ^2^ Department of Otolaryngology, Head and Neck Surgery, School of Medicine and Health, Technical University of Munich (TUM), Munich, Germany; ^3^ California Northstate University College of Medicine, Elk Grove, CA, United States; ^4^ Institute of Regenerative Biology and Medicine, Helmholtz Zentrum München, Munich, Germany; ^5^ Charité – Universitätsmedizin Berlin, Corporate Member of Freie Universität Berlin, Humboldt-Universität zu Berlin, Berlin Institute of Health, Department of Oral and Maxillofacial Surgery, Berlin, Germany

**Keywords:** bioengineered skin grafts, autologous skin grafting, skin deficits, plastic surgery, dermatologic surgery, human skin substitute

## Abstract

The reconstruction of complex skin defects challenges clinical practice, with autologous skin grafts (ASGs) as the traditional choice due to their high graft take rate and patient compatibility. However, ASGs have limitations such as donor site morbidity, limited tissue availability, and the necessity for multiple surgeries in severe cases. Bioengineered skin grafts (BSGs) aim to address these drawbacks through advanced tissue engineering and biomaterial science. This study conducts a systematic review to describe the benefits and shortcomings of BSGs and ASGs across wound healing efficacy, tissue integration, immunogenicity, and functional outcomes focusing on wound re-epithelialization, graft survival, and overall aesthetic outcomes. Preliminary findings suggest ASGs show superior early results, while BSGs demonstrate comparable long-term outcomes with reduced donor site morbidity. This comparative analysis enhances understanding of bioengineered alternatives in skin reconstruction, potentially redefining best practices based on efficacy, safety, and patient-centric outcomes, highlighting the need for further innovation in bioengineered solutions.

## Introduction

The management of large cutaneous defects arising from burns, traumatic injuries, chronic wounds, skin malignancies, and surgical interventions presents a multifaceted challenge in reconstructive surgery (Vecin and Kirsner, 2023). Autologous skin grafts (ASGs), which involve transplanting the patient’s own skin to the wound site, are commonly employed due to their reliable integration and low rejection rates. However, in cases of extensive injuries or limited donor tissue availability, ASGs may not be feasible (Kianian et al., 2023). Such limitations, including significant donor site morbidity, scarcity of available donor tissue in large injuries, and variable aesthetic results—particularly in full-thickness grafts—necessitate exploring alternative grafting options (Schlottmann et al., 2021).

**FIGURE 1:**
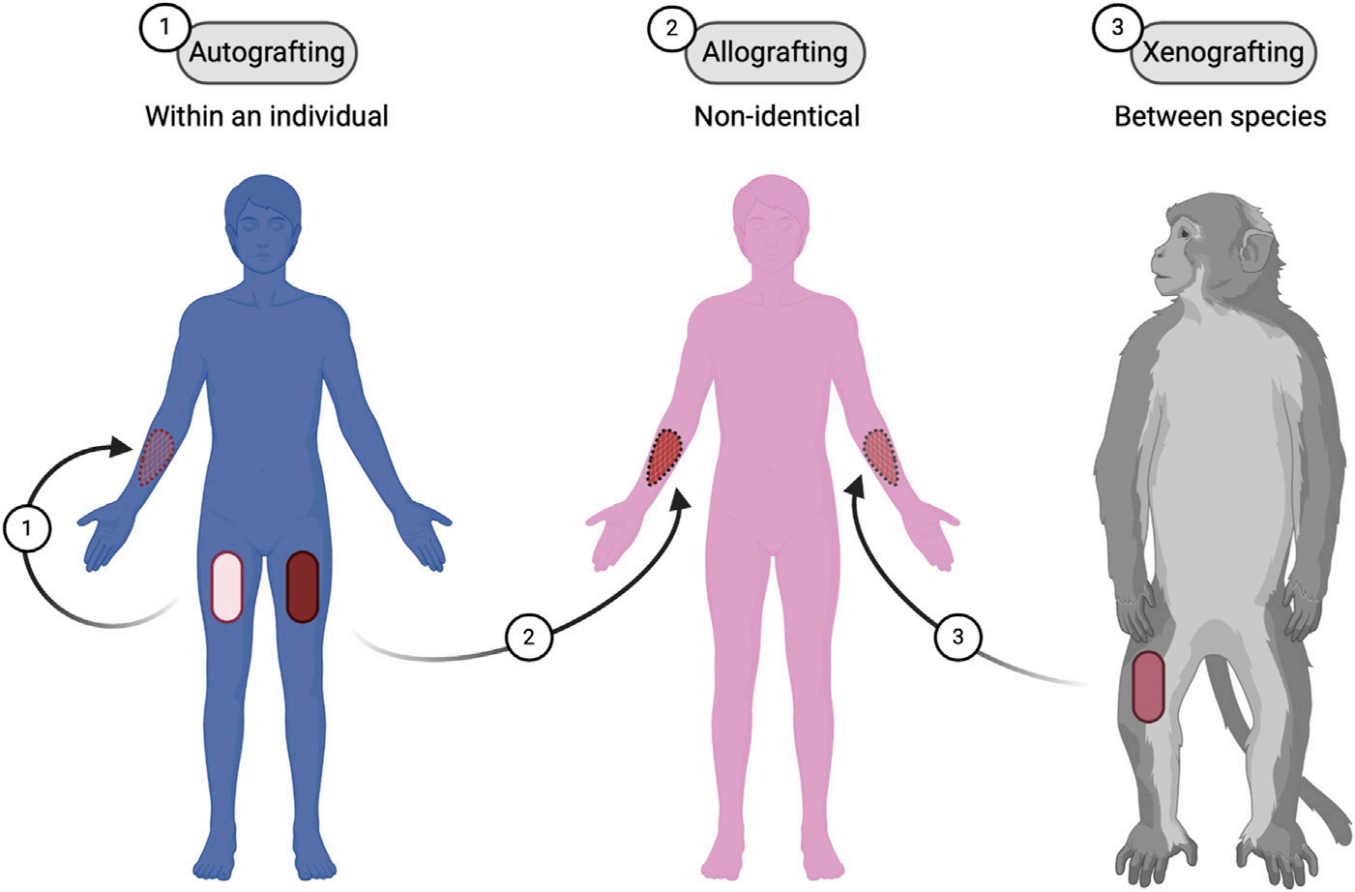
Types of skin grafting techniques. Three different types of skin grafting: (1) Autografting, where the graft is taken from the same individual; (2) Allografting, where the graft is from a non-identical donor of the same species; and (3) Xenografting, where the graft is from a different species. Figure created using BioRender, Toronto, ON, Canada.

**FIGURE 2:**
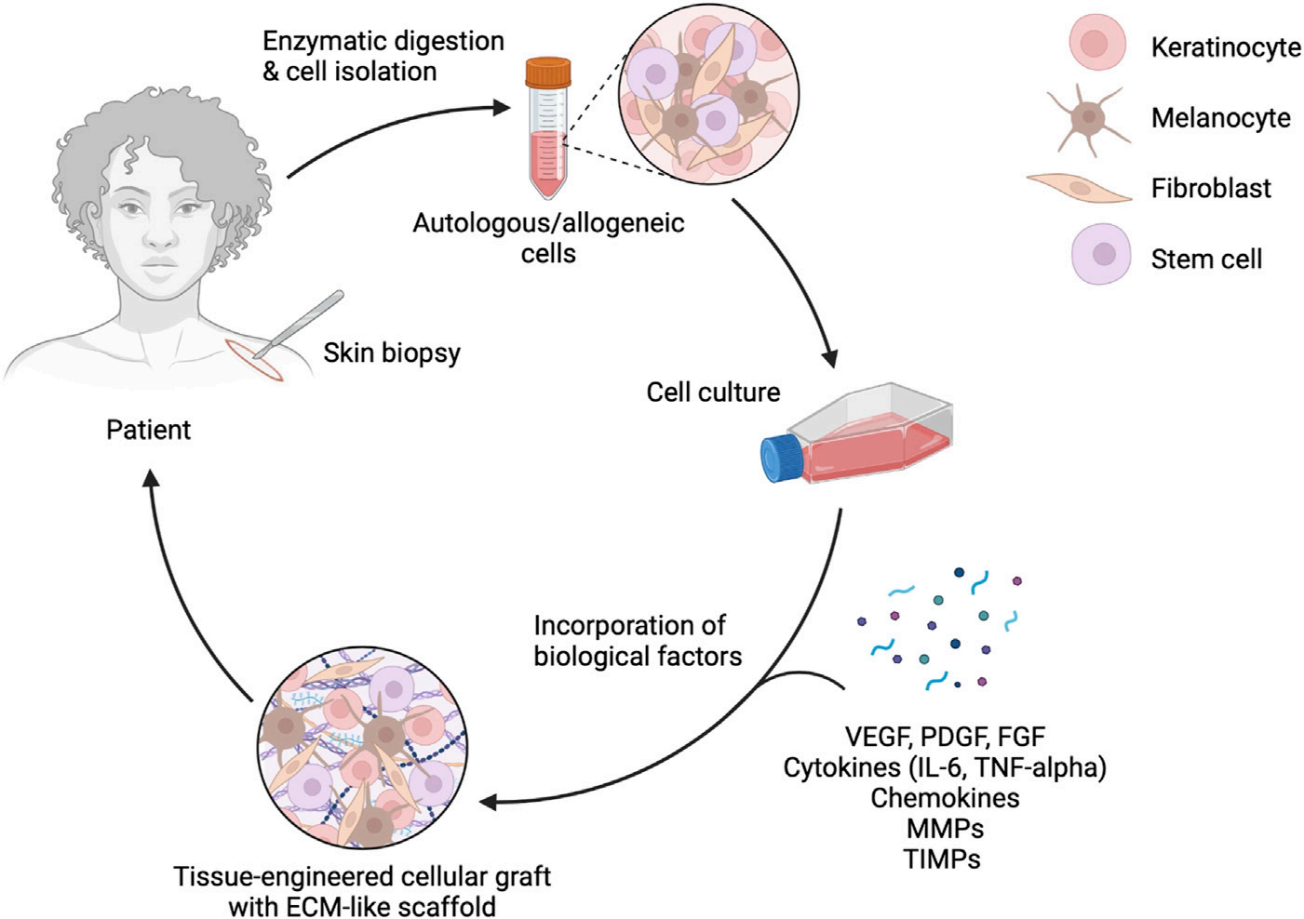
Enzymatic digestion and cell isolation for bioengineered skin grafts. This diagram illustrates the process of enzymatic digestion and cell isolation from a skin biopsy, followed by cell culture and incorporation of biological factors such as VEGF, PDGF, FGF, cytokines, chemokines, MMPs, and TIMPs. The final product is a tissue-engineered cellular graft with an ECM-like scaffold, ready to be applied back to the patient. Figure created using BioRender, Toronto, ON, Canada.

Bioengineered skin grafts (BSGs) emerge as a promising solution to these challenges. These grafts, derived from advancements in tissue engineering, regenerative medicine, and materials science, are designed to mimic the structure and function of native skin. BSGs consist of biocompatible scaffolds that support cellular proliferation and tissue regeneration (Han et al., 2020) (Figures 1, 2). They can be categorized into acellular and cellular types, with acellular grafts providing a structural framework for host cell infiltration, and cellular grafts incorporating specific cell types, such as keratinocytes and fibroblasts, to enhance tissue integration and regenerative capacity. Additionally, BSGs are often augmented with bioactive molecules, including cytokines and growth factors like transforming growth factor-beta (TGF-β), vascular endothelial growth factor (VEGF), platelet-derived growth factor (PDGF), and fibroblast growth factor (FGF), to further stimulate regenerative processes (Debels et al., 2015).

Despite their potential, BSGs face certain limitations that impact their widespread adoption. Key challenges include ensuring durability, integrating effectively with host tissues, managing immune responses, achieving aesthetic outcomes such as color matching, and optimizing vascularization. Overcoming these barriers is crucial for making BSGs a reliable option in clinical practice (Colazo et al., 2019). This analysis describes the clinical efficacy of ASGs and BSGs, focusing on outcomes such as wound closure, engraftment success, immunogenicity, functional recovery, and aesthetic satisfaction. Through a critical review of existing literature, we aim to elucidate the capabilities and constraints of both graft types, providing insights to guide future clinical strategies in reconstructive surgery. Ultimately, the goal is to deepen the understanding of these advanced technologies and optimize their application to improve patient outcomes in managing complex skin defects.

## Autologous skin grafting

**FIGURE 3:**
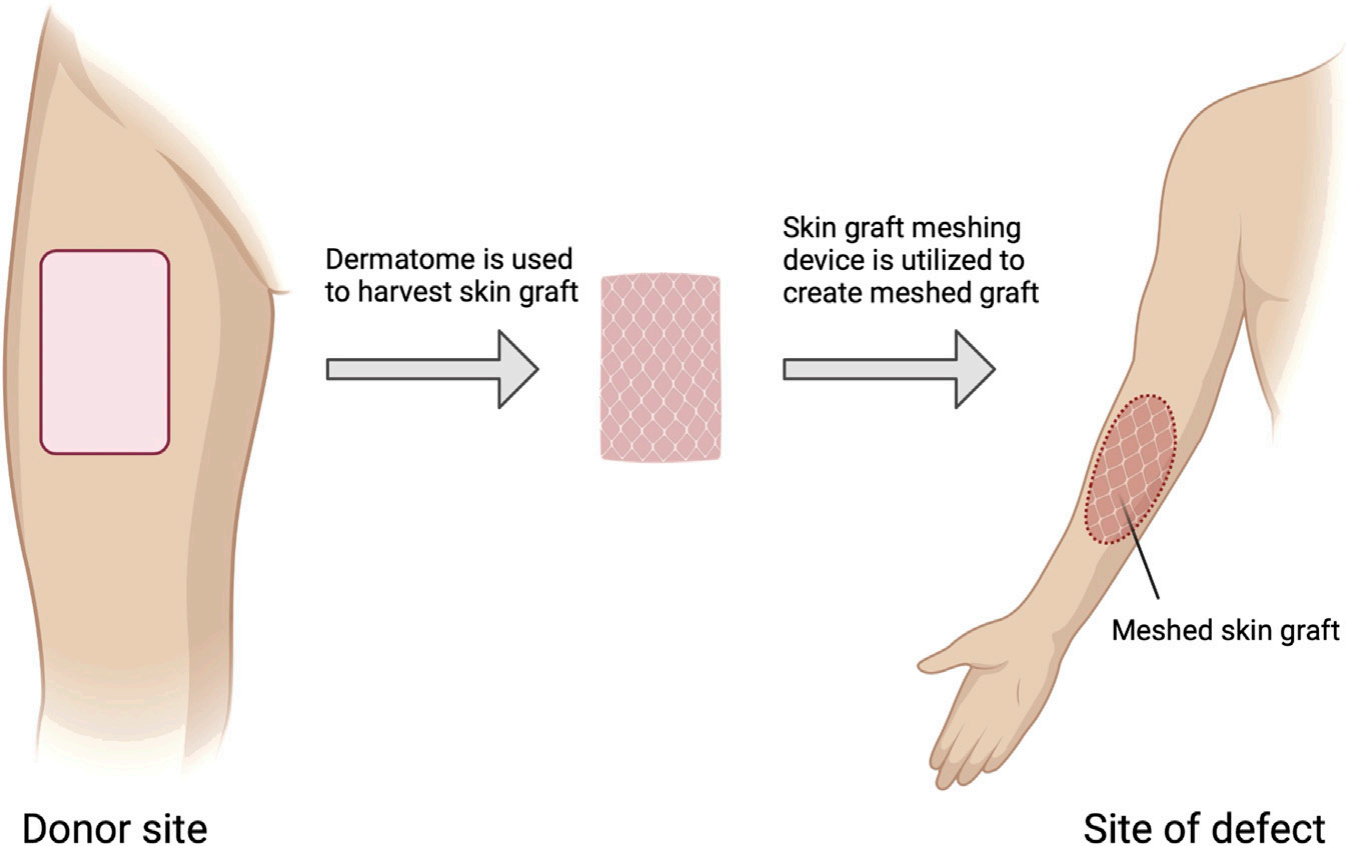
Autologous skin grafting process. Steps involved in autologous skin grafting, starting from the donor site where a dermatome is used to harvest the skin graft. The harvested skin is then meshed using a skin graft meshing device before being applied to the site of the defect. Figure created using BioRender, Toronto, ON, Canada.

ASGs are essential for repairing skin losses. The procedure involves transplanting skin from an uninjured donor site on the patient’s body to a damaged recipient site (Figure 3). This technique not only reduces the risk of immunogenic rejection but also utilizes the innate biological and immunological compatibilities of the patient’s own tissues, thereby optimizing the healing outcomes (Prohaska and Cook, 2024).

**FIGURE 4:**
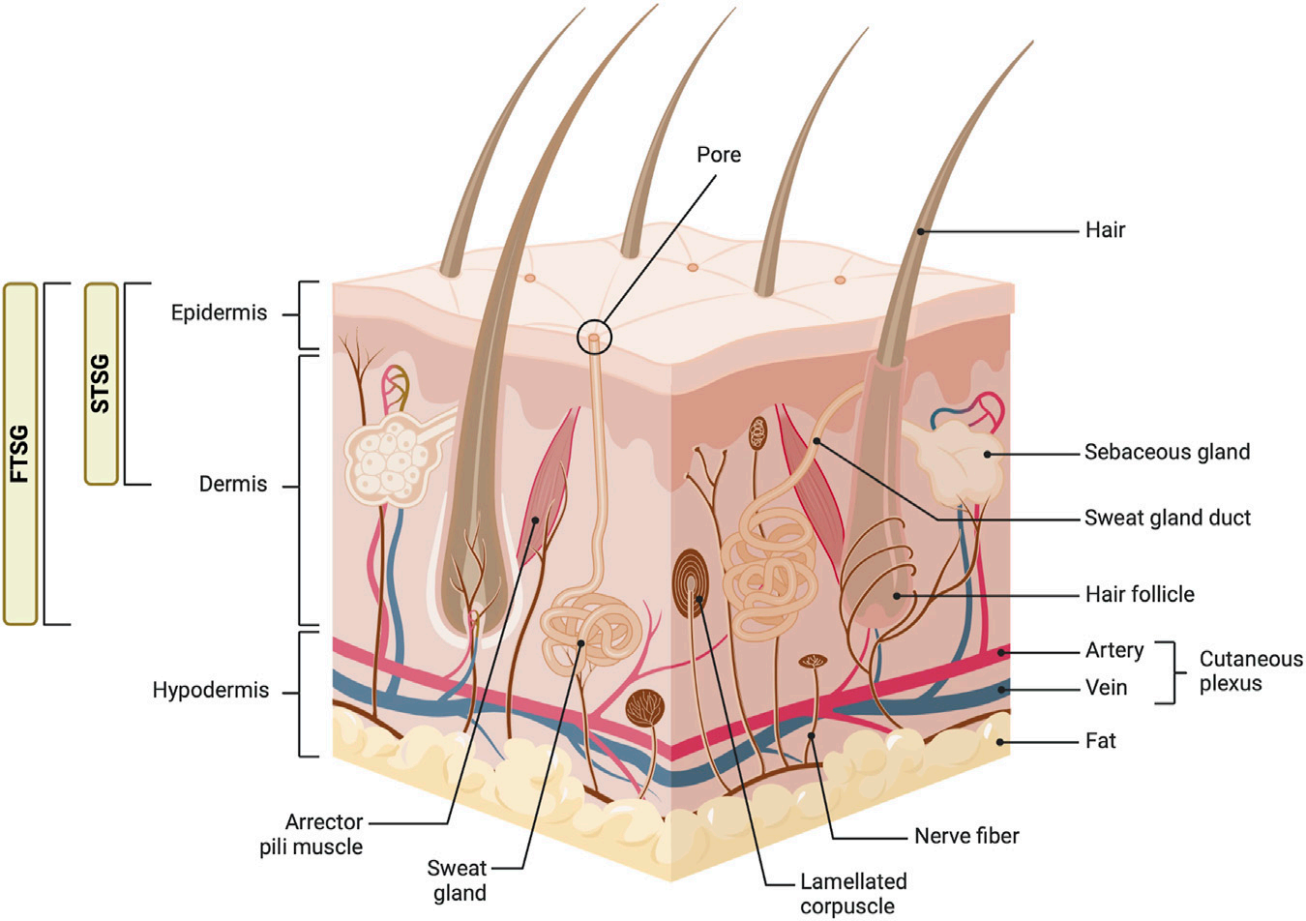
Anatomy of the skin and types of skin grafts. This diagram illustrates the detailed structure of the skin, including the epidermis, dermis, and hypodermis layers. Key components such as hair follicles, sebaceous glands, sweat glands, and various nerve fibers are labeled. The image also shows the distinction between full-thickness skin grafts (FTSG) and split-thickness skin grafts (STSG), highlighting the layers of skin each type includes. Figure created using BioRender, Toronto, ON, Canada.

ASGs encompass two primary types based on the depth of skin harvested: split-thickness skin grafts (STSGs) and full-thickness skin grafts (FTSGs) (Figure 4). STSGs, comprising the epidermis and a portion of the dermis, are favored for their enhanced engraftment success and expedited revascularization rates in covering large wound areas. This rapid revascularization is facilitated by the expression of various growth factors and cytokines, including VEGF and TGF-β, which promote angiogenesis and fibroblast migration (Braza and Fahrenkopf, 2024). However, their reduced depth of dermal elements can lead to suboptimal aesthetic results, including an increased risk of contraction and scarring. This is particularly evident in areas where skin elasticity and cosmetic appearance are crucial, such as the face or hands. Additionally, the thinner nature of STSGs may result in a less robust vascular supply, potentially impacting long-term graft survival (Braza and Fahrenkopf, 2024).

In contrast, FTSGs, encompassing both the epidermis and the entire dermis, are preferred for smaller, more cosmetically sensitive areas requiring superior tissue match and durability. The inclusion of the full dermal layer in FTSGs supports a richer presence of native skin appendages and a more robust extracellular matrix, leading to superior aesthetic results and reduced contraction (Osman and Emara, 2018). Nonetheless, ensuring the survival of FTSGs presents challenges due to the need for rapid and comprehensive revascularization to prevent necrosis of the transplanted tissue. Additionally, the thicker nature of FTSGs requires a more robust vascular connection for successful graft survival, making them more suitable for smaller, localized defects where adequate vascularization is achievable (Serra et al., 2017).

The immunological response to ASGs is less intense than that observed with allogeneic grafts, primarily due to the absence of foreign antigenic disparities (Benichou et al., 2011). However, surgical trauma during harvesting and grafting initiates a cascade of inflammatory responses mediated by cytokines such as interleukin-1 (IL-1), interleukin-6 (IL-6), and tumor necrosis factor-alpha (TNF-α), along with chemokines (Eming et al., 2007). These molecules modulate inflammation, wound healing, and subsequent remodeling phases (Ridiandries et al., 2018). Matrix metalloproteinases (MMPs), regulated by tissue inhibitors of metalloproteinases (TIMPs), are crucial in extracellular matrix remodeling at the wound site, facilitating necessary alterations for wound closure and graft integration. Proper balance between MMPs and TIMPs is essential to prevent excessive matrix degradation, which could compromise graft stability and aesthetic outcomes (Cabral-Pacheco et al., 2020).

Further, if the area requiring coverage is too large to be effectively addressed with an ASG, alternative reconstructive techniques may need to be considered. In such cases, where the deficit exceeds the available donor tissue or where the donor sites themselves are limited, surgeons may explore other options such as tissue expansion, tissue flaps, MEEK Micrografting, or bioengineered skin substitutes (Niermeyer et al., 2020; Kalaskar et al., 2016).

Tissue expansion involves gradually stretching adjacent healthy skin to generate additional tissue for coverage of the defect, which can take several weeks to months. This technique allows for the creation of larger skin flaps, thereby expanding the available donor area (Wagh and Dixit, 2013). This method, while effective in creating larger skin flaps and expanding the available donor area, can be time-consuming and uncomfortable for patients due to the prolonged stretching process and the presence of the expander. Additionally, tissue expansion carries risks of complications such as infection, hematoma, and tissue necrosis, which may necessitate further surgical interventions. Furthermore, tissue expansion often leaves noticeable scars at both the donor and recipient sites, impacting the overall cosmetic outcome (Gao et al., 2023; Xu et al., 2023).

MEEK micrografting offers a means to cover larger areas with less donor skin by expanding a small autologous graft up to nine times. This technique sections the donor skin into uniform micro-islands and applies them to the wound bed with specialized adhesive gauze, maintaining spacing for efficient re-epithelialization. Although MEEK micrografting can reduce the extent of required donor sites, it may increase infection risk in exposed areas between grafts and requires careful wound bed preparation to optimize graft success (Meek, 1958; Medina et al., 2016).

## Bioengineered skin grafts

Bioengineered skin substitutes offer a promising option for large-area coverage where traditional grafting techniques may be inadequate. These substitutes typically consist of synthetic or biologically derived materials that mimic the structure and function of native skin. They may be seeded with patient-derived cells or growth factors to enhance tissue regeneration and integration (Xu et al., 2023). While bioengineered skin substitutes continue to undergo refinement and optimization, they represent a valuable adjunct to traditional grafting techniques in cases of extensive skin loss. These grafts are primarily designed to address the limitations of traditional autologous and allogeneic grafts by providing off-the-shelf availability and reducing donor site morbidity while aiming to restore the physiological and mechanical properties of the skin following injury (Meek, 1958). The first skin substitute to receive FDA approval was Epicel^®^, followed by a wide array of products including spray-applied epidermal equivalents and composite scaffolds featuring extracellular matrix (ECM) components. These products are available on the market and categorized based on their cellular composition, an important factor in their biological similarity to natural skin (Medina et al., 2016). The two predominant types of bioengineered skin grafts include acellular grafts and cellular grafts.

### Acellular grafts

**FIGURE 5:**
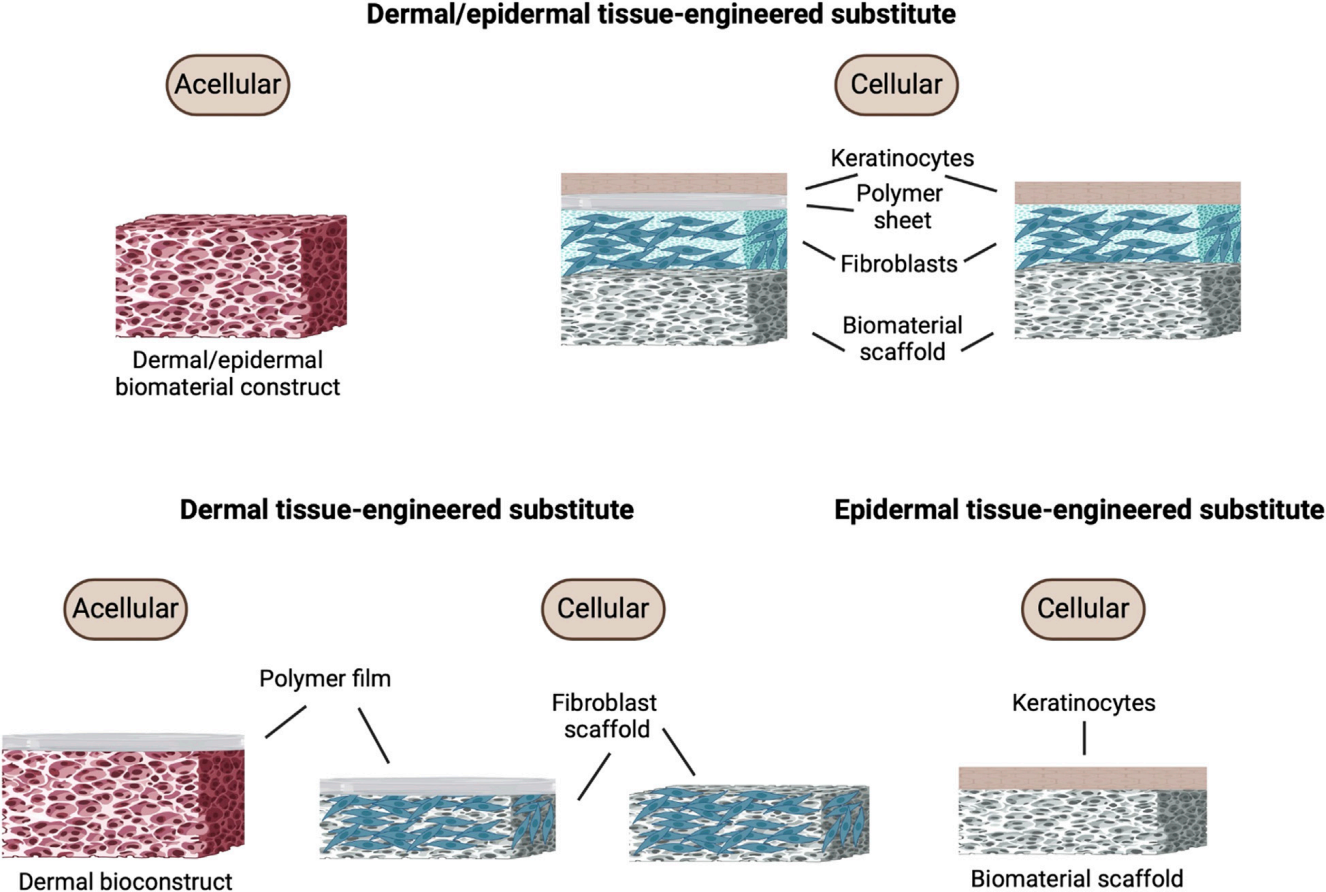
Bioengineered skin graft composition. Different types of tissue-engineered skin substitutes including acellular and cellular constructs for dermal/epidermal, dermal, and epidermal tissue-engineered substitutes. Each construct comprises different components such as keratinocytes, polymer sheets, fibroblasts, and biomaterial scaffolds. Figure created using BioRender, Toronto, ON, Canada.

Acellular grafts consist of biocompatible, biodegradable scaffolds made from natural or synthetic polymers such as collagen, fibrin, and polyglycolic acid (Figure 5). These materials are integral to tissue engineering due to their ability to support the body’s own healing mechanisms (Schoukens, 2019). Collagen, the most abundant protein in the skin’s extracellular matrix, is particularly valued for its biocompatibility and role in promoting cellular adhesion and proliferation (Amirrah et al., 2022). Fibrin, another commonly used material, is essential in wound healing, serving not only as a scaffold but also actively participating in blood clot formation during the initial wound-healing phase (Al Kayal et al., 2022). Polyglycolic acid, a synthetic polymer, is appreciated for its controlled degradation rate, allowing for gradual absorption and replacement by native tissue (Trucillo, 2024).

These scaffolds are engineered to act as matrices that support the ingrowth of the patient’s cells, primarily by facilitating the migration and proliferation of fibroblasts and keratinocytes from the wound edges (Dickinson and Gerecht, 2016). However, due to the limited proliferative capacity of keratinocytes and fibroblasts, especially for larger grafts, researchers have explored alternative cell sources (Phua et al., 2021). To enhance scaffold performance, physical and chemical properties such as porosity, fiber alignment, and degradation rate are finely tuned to optimize cellular infiltration and growth. For example, highly porous scaffolds improve cell migration and nutrient diffusion, both crucial for effective tissue integration and regeneration (Lutzweiler et al., 2020).

Recent advancements have incorporated nanoparticles containing growth factors and antibiotics into acellular grafts, allowing controlled, gradual release to boost treatment efficacy (Lin et al., 2022). Similarly, nanomaterials designed to mimic natural tissue structure can promote the bonding of fibroblasts, keratinocytes, and epithelial cells, supporting re-epithelialization and angiogenesis (Bellu et al., 2021). For instance, electrospun scaffolds with a pore size of 100–200 μm have effectively supported fibroblast survival and maintenance *in vitro* (Rnjak-Kovacina and Weiss, 2011), while collagen-based electrospun scaffolds reduced wound contraction by 22% compared to freeze-dried scaffolds in murine models (Powell et al., 2008). Nanoparticles like silver and gold, known for their bactericidal properties, are also incorporated to improve biocompatibility and mechanical strength. Silver nanoparticles and gold nanoparticles at concentrations of 14.27 ppm have been shown to sustain murine 3T3 fibroblast and keratinocyte cultures for up to 14 days *in vitro* without toxicity (Akturk et al., 2016).

The interaction between these scaffolds and the biological environment is critical to their functionality (Loh and Choong, 2013). In addition to supporting cell adhesion and growth, acellular grafts must also modulate the local immune response to prevent chronic inflammation, which can impede healing. Scaffold designs that allow the gradual release of bioactive molecules, such as growth factors or cytokines, can further enhance healing by promoting angiogenesis and regulating immune responses (Russo et al., 2022; Wang F. et al., 2023).

For current acellular options, please refer to Table 1.

**TABLE 1:** Summary of acellular and cellular bioengineered human skin substitutes, including primary uses, key features, and additional clinical considerations.

Product	Primary Use	Key Features and Additional Information
Acellular		
Alloderm®	Reconstructive surgery	- Promotes vascularization
		- Particularly useful for abdominal and breast reconstructive surgeries
Biobrane®	Burns, wound care	- Controls bacterial growth
		- Conforms to surface irregularities
		- Porcine origin may limit acceptance due to associated risks
		- Removed after epithelialization
Integra®	Burns, reconstructive surgery	- Used with Recell® in a one-step process for skin regeneration
		- Supports faster recovery and reduced scarring
Matriderm®	Skin grafting	- Reduces hematoma risks with its hemostatic properties
		- Improves post-operative outcomes
OASIS®	Wound management	- Maintains extracellular matrix components in bioactive forms
		- Provides a natural scaffold for cellular infiltration and tissue growth
Permacol™	Surgical repair, soft tissue reconstruction	- Features crosslinking that guards against biological degradation
		- Enhances durability in the surgical repair of tissues
Suprathel®	Wound care, surgical recovery	- Anti-sepsis properties and controls bleeding effectively, though less suited for full-thickness wound healing
Terudermis®	Surgical repair, skin grafting	- Combines collagen and silicone
		- Often used with growth factors and cultured fibroblasts for enhanced healing alongside split-thickness skin grafts
NovoSorb™ BTM	Deep dermal or full thickness wounds	- Biodegradability reduces the need for surgical removal
		- Integration with tissue, encouraging the growth of new blood vessels and connective tissue
		- Used in various depths and extents of skin loss
		- Potentially reduces the formation of hypertrophic scars
Mucograft	Extraoral defects, periodontal surgery	- Mimics the natural architecture of connective tissue, facilitating cell migration and angiogenesis
		- Scaffold supports rapid vascularization and integration with surrounding tissues, leading to quicker healing times
Cellular		
Apligraf®	Ulcers	- Enhances healing by producing cytokines and growth factors
		- Mimicks native skin functionality
Bioseed-S®	Chronic venous leg ulcers	- Demonstrates superior effectiveness in treating chronic venous leg ulcers
		- Offers about 50% greater effectiveness compared to standard treatments
Dermagraft®	Wound care	- Provides a collagen-rich matrix that supports active fibroblast functioning for advanced wound care
Epidex™	Skin autografts, wound closure	- Effective in facilitating the healing of split-thickness skin autografts and managing chronic venous leg ulcers
EPIBASE®	Wound management	- Involves direct application of patient-derived cell suspensions to wounds
		- Supports natural healing processes
Hyalograft 3D®	Full-thickness wounds, burn care	- First full-thickness autologous skin substitute used with Laserskin® in a complex grafting system to enhance healing
OrCel®	Burn care, reconstructive surgery	- Noted for reduced scarring and high efficacy in treating burns and other complex wounds
Transcyte®	Wound care, burn treatment	- Speeds epithelial renewal
		- Reduces dressing changes
		- Carries risks of inflammation and rejection due to its content derived from human foreskin fibroblasts

### Cellular grafts

Cellular grafts represent a more complex form of bioengineered skin substitutes, where the scaffolds are pre-seeded with specific types of cells, such as keratinocytes, fibroblasts, melanocytes, and stem cells (Figure 5). These cells may be autologous, derived from the patient to reduce the risk of immunogenic reactions, or allogeneic, sourced from donors, which can offer different functional benefits but with an increased risk of immune rejection (Oualla-Bachiri et al., 2020; Sierra-Sánchez et al., 2021). Studies involving adipose-derived stem cells (ADSCs) encapsulated in fibrin-chitosan matrices have demonstrated consistent release of these factors, which aid in the angiogenic process essential for healing (Kaur et al., 2019). The presence of ADSCs has also been shown to enhance collagen deposition and fibroblast homing, significantly augmenting neovascularization (Mazini et al., 2020).

The scaffold structures in cellular grafts are critical as they provide the necessary environment for cell attachment, growth, and maturation. Techniques like electrospinning are employed to create fibrous meshes that closely mimic the natural extracellular matrix, thus providing an optimal environment for cells (Ahmed et al., 2019). Advanced cell culture techniques, including the use of bioreactors, are often required to cultivate these cells on the scaffolds under controlled conditions, facilitating the development of the desired tissue structures before implantation (Anjum et al., 2022; Ahmed et al., 2019).

From an immunological perspective, managing the immune response is crucial for the success of cellular grafts. Acellular constructs typically provoke fewer immune reactions due to their lack of foreign cells, but cellular constructs, particularly those with allogeneic cells, need to balance functionality with the risk of immune rejection. This may necessitate the use of immunosuppressive agents or the integration of immune-modulating substances into the scaffold to minimize potential rejection (Sierra-Sánchez et al., 2021; Zakrzewski et al., 2014).

Cellular grafts also heavily rely on angiogenesis for successful integration into the host tissue. The rapid establishment of a vascular network is essential to meet the metabolic demands of the newly forming tissue (Lovett et al., 2009). This is often facilitated by incorporating angiogenic factors, such as VEGF and FGF, directly into the graft. Additionally, cytokines such as IL-6 and TNF-alpha, along with various chemokines, play critical roles in modulating inflammation and promoting the recruitment and activation of cells necessary for tissue repair and regeneration (Veith et al., 2019).

Matrix metalloproteinases (MMPs) are significant in the remodeling phase, aiding in the degradation and reorganization of the extracellular matrix to accommodate new tissue growth. However, uncontrolled MMP activity can lead to excessive degradation, potentially undermining graft stability. Including tissue inhibitors of metalloproteinases (TIMPs) in the graft design can help regulate MMP activity, ensuring a balanced remodeling process that supports long-term graft integration and function (Lee and Kim, 2022).

For current cellular options, please refer to Table 1.

### Tri-layered substitutes

As opposed to the acellular grafts, tri-layered substitutes are advanced skin substitutes designed to mimic the natural structure of skin, and they typically have three distinct layers (epidermal, dermal, and hypodermal), while acellular grafts only have the dermal component. Current commercial skin substitutes, while offering promise in wound healing, fall short in replicating the trilaminar architecture of native skin (Savoji et al., 2018). The hypodermis plays a pivotal role in facilitating vascularization, adipose tissue deposition, and sensory functions, underscoring its indispensability in skin regeneration processes (Jäger et al., 2024). Of particular concern is the absence of an authentic hypodermal layer in existing substitutes, impeding their ability to fully address the requirements of full-thickness wound healing (Jäger et al., 2024; Nourian Dehkordi et al., 2019).

In response to this, research efforts have been directed toward the development of tri-layered skin substitutes that aim to mimic the native tissue architecture. These tri-layered constructs typically encompass an epidermal layer, a dermal layer, and a subcutaneous layer (Zimoch et al., 2021). However, achieving fidelity to the hypodermal layer remains a challenge, often necessitating the incorporation of synthetic components to simulate its structural and functional attributes. While significant progress has been made, with some tri-layered substitutes demonstrating promising results in preclinical and clinical studies, the quest for a truly biomimetic hypodermal layer persists (Hong et al., 2023). Synthetic materials such as biocompatible polymers and hydrogels are frequently used to fabricate the hypodermal analog, with efforts focused on optimizing their mechanical properties, porosity, and bioactivity to enhance their compatibility with host tissues (Revete et al., 2022).

An emerging strategy involves the integration of basement membrane analogs within the tri-layered constructs to emulate the supportive matrix that underlies the epidermis and facilitates cell adhesion, migration, and differentiation. By incorporating elements of the native extracellular matrix architecture, these basement membrane mimetics serve to enhance the structural integrity and functionality of the skin substitutes, fostering more robust tissue regeneration processes (Jain et al., 2022). Despite these advancements, challenges persist in achieving integration and long-term stability of tri-layered skin substitutes within the wound microenvironment. Factors such as host immune response, vascularization, and mechanical stresses impose constraints on the clinical translation of these constructs (Oualla-Bachiri et al., 2020; Zhang et al., 2023), necessitating further research into strategies for promoting tissue integration, minimizing immunogenicity, and enhancing overall therapeutic efficacy.

## Considerations for BSG design

### Immunological considerations

**FIGURE 6:**
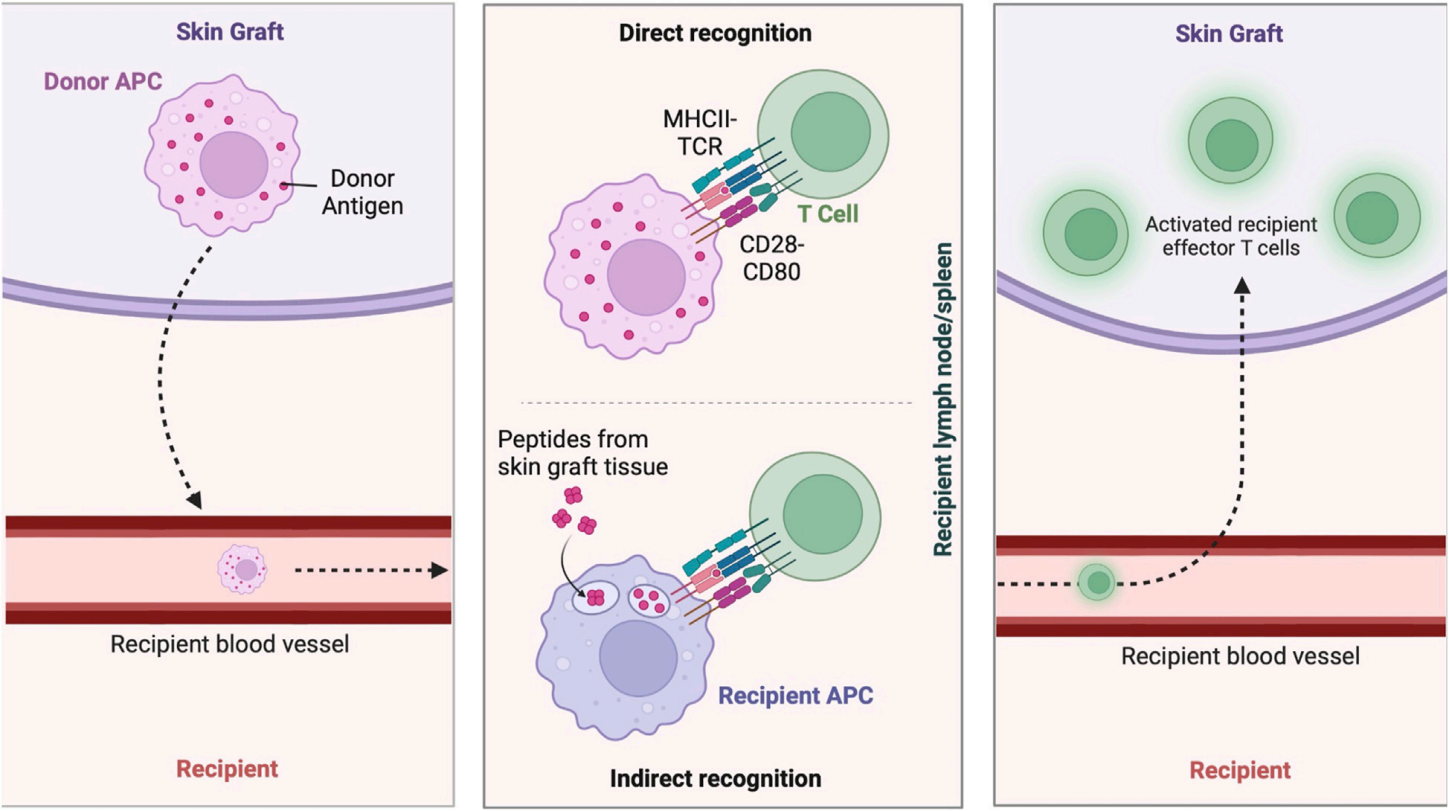
Immune response to skin grafts. Immune mechanisms involved in skin graft rejection and acceptance, including donor antigen presentation by donor APCs, direct recognition by T cells, and indirect recognition by recipient APCs, leading to the activation of effector T cells in the recipient. Figure created using BioRender, Toronto, ON, Canada.

Bioengineered grafts present complex challenges and require careful consideration of immunological compatibility to ensure successful integration and functionality (Oli et al., 2022). Immunological considerations profoundly shape the development and application of bioengineered grafts, necessitating a meticulous understanding of cellular interactions, signaling pathways, and molecular mediators to optimize graft integration and circumvent immunological barriers (Figure 6). Allogeneic (Benichou et al., 2011) and xenogeneic (Gock et al., 2004) bioengineered skin grafts, with their foreign cellular components, are particularly prone to eliciting robust immune responses leading to rejection (Dixit et al., 2017). Xenogeneic grafts, marked by substantial antigenic disparities between donor and recipient species, evoke heightened immune responses through the activation of both innate and adaptive immune pathways (Zhou et al., 2013). This is primarily due to the immune system’s inherent capacity to distinguish self from non-self, triggering intricate cascades of cellular and molecular events aimed at eliminating these perceived foreign entities (Benichou et al., 2011; Dixit et al., 2017; Metcalfe and Ferguson, 2007).

To mitigate immunogenicity, the use of autologous cells has been explored. However, modifications to autologous cells for therapeutic purposes necessitate careful preservation of their immune compatibility. In regenerative medicine, a common strategy to achieve this involves genetic engineering approaches designed to attenuate immune activation (Martin et al., 2024). This includes the upregulation of immune-evasive molecules such as the expression of immune-evasive molecules like programmed death-ligand 1 (PD-L1) or the downregulation of major histocompatibility complex (MHC) class I molecules to prevent sensitization and subsequent rejection (Zakrzewski et al., 2014). Strategies leveraging genetic engineering to modify these immunological properties or employing immunosuppressive agents locally are also pursued to reduce graft rejection (Kauke-Navarro et al., 2023; Arruda et al., 2009).

The modulation of antigen presentation mechanisms is a pivotal strategy to mitigate graft rejection. Specifically, the direct pathway involves the presentation of intact donor MHC molecules by antigen-presenting cells (APCs) within the graft tissue to recipient T cells, eliciting their activation and subsequent immune response against the transplant (Afzali et al., 2008). The indirect pathway entails the processing and presentation of donor antigens by recipient APCs via self-MHC molecules, instigating an immune response against the graft (Game and Lechler, 2002). Additionally, the semi-direct pathway, which combines elements of both direct and indirect pathways, involves the direct engagement of recipient T cells by donor APCs within the graft, further amplifying the immune cascade against the allograft (Afzali et al., 2008).

Critical mediators orchestrating the immune response include cytokines, chemokines, and interleukins (ILs), delineating the spatiotemporal dynamics of immune cell activation and function (Altan-Bonnet and Mukherjee, 2019). Notably, interleukins (ILs) such as IL-1, IL-6, and IL-8 foster an inflammatory microenvironment conducive to immune cell recruitment and activation (Vasalou et al., 2023). Chemokines, including CCL2, CXCL10, and CXCL12, play pivotal roles in orchestrating leukocyte trafficking and homing to the graft site, thereby modulating immune responses (Nelson and Krensky, 2001; Olson and Ley, 2002). Additionally, cytokines such as TNF-α, Interferon-gamma (IFN-γ), and IL-2 exert multifaceted effects on immune cell activation, proliferation, and effector functions, thereby shaping the magnitude and duration of the immune response against the graft (López-García and Castro-Manrreza, 2021). These interactions involve key signaling pathways such as nuclear factor-kappa B (NF-κB), activated by pro-inflammatory cytokines to upregulate immune genes, and JAK/STAT, crucial for the signaling of cytokines like IFN-γ and IL-6 that drive immune cell differentiation and function (Hu et al., 2021).

### Angiogenesis

Angiogenesis, the formation of new blood vessels, is essential for the viability and success of bioengineered skin grafts (avascularized grafts). Establishing a functional microvascular network within the graft is crucial for nutrient and oxygen delivery, cellular metabolism, and overall integration with host tissue (Phua et al., 2021; Laschke et al., 2006). This complex process depends on the interplay between various cell types, signaling pathways, and scaffold properties that facilitate vascularization (Akbarian et al., 2022; Lamalice et al., 2007). Endothelial cells, which form the lining of blood vessels, drive angiogenesis through proliferation, migration, and tube formation. Their function is supported by perivascular cells, such as pericytes, which stabilize nascent vessels and promote vessel maturation (Zhao and Chappell, 2019). This controlled angiogenic environment optimizes conditions for graft integration by enabling early vascular support and reducing inflammatory disruptions. Together, these cells establish a microvascular network that can sustain the graft’s metabolic needs (Lovett et al., 2009).

The scaffold material itself also plays a vital role in angiogenesis (Chan and Leong, 2008). Scaffold properties, such as mechanical stiffness, porosity, and biochemical cues, are designed to support cellular infiltration and the maturation of newly formed blood vessels. Materials that allow for gradual biodegradation are especially effective, as they provide temporary structural support while promoting the formation and integration of natural vasculature (Muzzio et al., 2021). Bioprinting technology enhances angiogenesis by incorporating vascular cells directly into the graft scaffold. For example, Baltazar et al. used bioprinting with bio-inks infused with endothelial cells, pericytes, fibroblasts, and keratinocytes, enabling natural vascular network assembly *in vitro*. Upon implantation, these pre-vascularized constructs displayed immediate perfusion and sustained vessel integrity, demonstrating bioprinting’s potential to facilitate early vascularization and improve graft survival (Baltazar et al., 2020).

Angiogenesis within these grafts is further enhanced by growth factors like vascular endothelial growth factor (VEGF), platelet-derived growth factor (PDGF), and fibroblast growth factor (FGF). These factors stimulate endothelial cell proliferation, migration, and vessel formation (Lamalice et al., 2007). Additionally, cytokines like IL-1, IL-6, and TNF-α coordinate both inflammatory and angiogenic responses within the graft environment, optimizing conditions for rapid and stable vascularization (Everts et al., 2023; Mahmoud et al., 2024). These signaling molecules activate pathways that increase vascular permeability, capillary sprouting, and vessel maturation, which are essential for integrating the graft with host tissue.

### Vascularization

Vascularization is crucial in the development of skin substitutes as it directly affects their complete biological function. Angiogenesis refers to the formation of new blood vessels from pre-existing ones, whereas vascularization is the process of developing an entire network of blood vessels in a tissue or organ, including both angiogenesis and vasculogenesis (Nitzsche et al., 2022). Without proper vascularization, a skin substitute cannot adequately receive oxygen and nutrients from the surrounding wound bed (Shahin et al., 2020; Rademakers et al., 2019). The physiological processes following a skin injury highlight the necessity for advancing research into pre-vascularized skin substitutes (Oualla-Bachiri et al., 2020). After an injury, an integrated healing response begins to restore the damaged tissue through a sequence of phases: hemostasis/inflammation, proliferation, and remodeling. Initially, platelets adhere to exposed ECM components, such as collagen and von Willebrand factor, triggering a cascade that leads to clot formation and activation of inflammatory cells like macrophages and neutrophils. These cells amplify the inflammatory response and facilitate phagocytosis to clean the wound area, setting the stage for healing (Diller and Tabor, 2022).

During the proliferation phase, heightened blood perfusion to the wound site serves as a critical driver for tissue regeneration by facilitating the delivery of essential nutrients, growth factors, and cellular constituents (Neishabouri et al., 2022). This enhanced blood flow not only replenishes oxygen and metabolic substrates but also acts as a conduit for the transportation of signaling molecules pivotal for orchestrating the proliferative response (Neishabouri et al., 2022; Landén et al., 2016). Among the diverse cellular populations recruited to the wound milieu, endothelial cells, keratinocytes, and fibroblasts emerge as key protagonists in the regenerative process. Endothelial cells, crucial for angiogenesis initiation, respond to cues from the microenvironment such as growth factors and chemokines by proliferating and migrating to form nascent blood vessels (Ridiandries et al., 2018; Rodrigues et al., 2019). Keratinocytes, the predominant cell type in the epidermis, undergo rapid proliferation to replenish the epithelial barrier and facilitate wound closure. Fibroblasts, pivotal in ECM synthesis and remodeling, proliferate and deposit collagen to provide structural support for tissue regeneration (Diller and Tabor, 2022; Xue and Jackson, 2015).

Cultured epithelial substitutes (CESs), which are predominantly composed of keratinocytes, have shown varied graft survival rates across different studies. Some studies indicated that when used alone, without a dermal component, the CESs had a graft survival rate of 63.6% (Rennekampff et al., 1997). However, other studies have demonstrated that outcomes improve when CESs are combined with other treatments like meshed split-thickness skin grafts (Teepe et al., 1990; Blight et al., 1991). A study highlighted the benefits of integrating collagen and elastin with autologous CESs prior to the application of standard autograft treatments. This approach resulted in a higher rate of epithelization, where the combination treatment achieved a 71% success rate compared to 67% with autografts alone. The Patient and Observer Scar Assessment Scale (POSAS) scores also improved, recording 14.2 ± 7.2 for the combined treatment versus 18.4 ± 10.2 for autografts alone (Gardien et al., 2016). Composite skin substitutes (CSSs), which combine both keratinocytes and fibroblasts, have been shown to cover up to 70% of wound areas effectively in some cases, showing their capability to reduce healing time and improve aesthetic outcomes compared to traditional treatments.

In cases of full-thickness wounds, this normal healing response is impaired or absent, leading to a physiological crisis in non-prevascularized skin substitutes due to insufficient nutrient and oxygen supply, severely affecting healing outcomes. Creating a prevascularized graft fosters integration with the host’s blood vessels and enhances the viability of the skin structure, ultimately leading to a higher success rate of implantation (Klar et al., 2014). Chen et al. demonstrated that prevascularized human MSC sheets, when transplanted with autologous split-thickness skin grafts, expedite wound healing in rat models of full-thickness skin wounds (Chen et al., 2017). Paralleling these findings, Miyazaki et al. showcased promising outcomes in accelerated wound healing through implantation of an innovative 3D scaffold-free pre-vascularized alternative in immuno-deficient mouse models (Miyazaki et al., 2019). In contrast to non-vascularized tissue constructs, which exhibited inadequate collagen deposition likely due to insufficient blood supply, the pre-vascularized substitute showed no signs of epidermal sloughing (Miyazaki et al., 2019). Thus, it is evident that a greater number of vessels are crucial for enhancing the maintenance and survival of the graft, particularly in the initial stages. Intriguingly, disparities were observed 7 days post-grafting. Blood vessels were solely present along the circumferential border of the non-vascularized graft, whereas the pre-vascularized graft displayed well-perfused blood vessels throughout. Apart from promoting strong graft adherence, the pre-vascularized skin substitute enables rapid perfusion, thereby augmenting collagen deposition and increasing dermal thickness (Miyazaki et al., 2019).

The pre-vascularization strategy is particularly promising as it establishes a direct blood supply through pre-formed vessels, enhancing skin graft survival and integration. This approach also accelerates wound healing, increases collagen deposition, and stabilizes epidermal homeostasis, reducing contraction and improving healing markers (Chen et al., 2017). In the context of vascularization, members of the VEGF family, comprising VEGF-A, VEGF-B, VEGF-C, and VEGF-D, serve as regulators of angiogenesis, exerting potent pro-angiogenic effects by promoting endothelial cell proliferation, migration, and vessel permeability (Holmes and Zachary, 2005). Similarly, FGF and TGF-β play pivotal roles in modulating vascular remodeling processes and endothelial cell behavior. In particular, TGF-βacts as a potent inducer of angiogenesis by promoting endothelial cell proliferation and vessel stabilization (Ma et al., 2020). The interplay between these angiogenesis inductors and inhibitors dictates the dynamics of vascularization within the tissue-engineered construct, thereby influencing graft integration, functionality, and long-term outcomes (Saberianpour et al., 2018). Strategies leveraging autologous cell sources, such as the stromal vascular fraction (SVF) derived from adipose tissue, offer promising avenues for enhancing vascularization and graft performance in tissue engineering applications (Koh et al., 2011). By harnessing the regenerative potential of endogenous cell populations and angiogenic factors, pre-vascularization strategies hold considerable promise for improving the clinical efficacy and translational potential of tissue-engineered constructs in regenerative medicine. Emerging technologies and approaches such as 3D scaffolds and microfluidic systems continue to evolve, aiming to create organized and functional vascular networks that closely mimic natural tissue characteristics. These advancements highlight the ongoing need to develop effective vascularization strategies that can significantly impact the success of skin substitutes in clinical applications, ultimately improving patient outcomes in skin repair and regeneration (Lovett et al., 2009; Yeo et al., 2024).

## Autologous skin grafts vs. bioengineered skin substitutes

Compared to conventional autologous skin grafts, bioengineered skin substitutes offer several advantages that address the limitations of traditional grafting techniques. These grafts, produced *in vitro* without relying on donor skin, overcome the challenge of limited donor sites and reduce patient morbidity (Kianian et al., 2023). Additionally, bioengineered grafts can be customized by incorporating specific growth factors, cytokines, and extracellular matrix components to promote angiogenesis, tissue regeneration, and better integration with host tissue (Oualla-Bachiri et al., 2020). The inclusion of ECM components such as fibronectin, collagen, and laminin provides structural support and essential biochemical cues that guide endothelial cell behavior, aiding the formation and maturation of new blood vessels within the graft (Spang and Christman, 2018).

## Applications across *in-vitro*, *in-vivo*, pre-clinical, and clinical settings

### *In-vitro* and *in-vivo*

In the development of optimized, biocompatible dermal matrices that promote wound healing and functional restoration, researchers have explored innovative approaches involving bioactive molecules and cellular integration. Mineo et al. (2013) investigated a hyaluronic acid-collagen artificial dermis enriched with epidermal growth factor (EGF), testing its effects in both *in vitro* and *in vivo* settings. Initial *in vitro* assessments demonstrated that EGF-enhanced dermis (Type II) significantly stimulated fibroblast activity, leading to elevated production of VEGF and hepatocyte growth factor (HGF), which are critical for wound repair and vascularization. Subsequent *in vivo* testing in a rat model with deep dermal burns confirmed that Type II dermis promoted angiogenesis and reduced inflammation more effectively than the EGF-free control (Type I), suggesting potential clinical applications for EGF-enriched dermal matrices in wound healing through targeted growth factor delivery and inflammation modulation.

Building on cell-based strategies, Wang Z. et al. (2023) investigated the use of epithelial stem cells (EpSCs) combined with acellular dermal matrix (ADM) and split-thickness skin grafts (STSGs) to improve vascularization in full-thickness wound healing. *In vitro* assays using EpSC-conditioned ADM supernatant showed enhanced angiogenesis in rat vascular endothelial cells, with RNA sequencing and tube formation assays confirming this effect. *In vivo*, EpSC-treated groups displayed improved graft survival, reduced wound contraction, and enhanced cosmetic outcomes. Transcriptome analysis indicated upregulation of key angiogenic pathways, including IL-17 and HIF-1 signaling, underscoring the potential of EpSCs to support vascularization in ADM-based wound care applications.

Addressing the challenge of pigmentation mismatch—particularly significant for patients with darker skin tones, Ng et al. (2018) developed a 3D biomimetic dermal construct that supports the co-culture of fibroblasts, keratinocytes, and melanocytes. Using optimized culture conditions and 3D bioprinting, they achieved a porous structure mimicking natural skin’s hierarchy, which facilitated even distribution of melanin granules in the epidermal layer. This approach enabled the creation of naturally pigmented, full-thickness skin grafts, promising for clinical applications requiring color-matched skin replacements. However, re-pigmentation rates vary; some cases show results in 3–5 weeks, while others experience delays of up to 4 months. Compared to manually casted constructs, the bioprinted skin showed more uniform pigmentation after 39 days of *in vitro* culture (Ng et al., 2018). Supporting this effort, Harriger et al. (1995) demonstrated that “passenger” melanocytes, inadvertently transplanted alongside keratinocytes and fibroblasts, promoted spontaneous re-pigmentation in artificial skin grafts within 2 months. Additionally, the commercial product ReCell^®^, which uses autologous keratinocytes, melanocytes, and fibroblasts, showed varied re-pigmentation rates, with bioprinted constructs achieving more uniform pigmentation within 39 days, although efficacy decreased in patients over 30 years old.

Mohd Hilmi et al. (2013) conducted foundational research on chitosan-based biomaterials to address full-thickness wound healing, particularly in radiation-damaged tissues. Recognizing the risk of scarring from incomplete reepithelialization, they explored both a chitosan dermal substitute and a chitosan skin substitute in irradiated rat models. The chitosan substitutes demonstrated superior reepithelialization rates and significantly smaller scar sizes compared to duoderm-treated controls, suggesting that chitosan may be a promising biomaterial for minimizing scarring and improving outcomes in complex wounds. Immunohistochemical analysis revealed human leukocyte antigen (HLA) expression on days 7, 14, and 21, confirming the presence and survival of human hair follicle stem cells and fibroblasts within the irradiated wounds. These findings suggest that chitosan-based dermal and skin substitutes hold promise as regenerative biomaterials in improving the healing and minimizing scar formation of full-thickness wounds, particularly those complicated by radiation damage.

Mahmoud and Salama (2016) contributed to this area by developing a chitosan/gelatin sponge enriched with vitamin C and cross-linked with tannin acid. This material accelerated healing in rabbit models and showed antibacterial properties against *E. coli* and *S. aureus* without toxicity, positioning it as a potential material for infection-prone wounds. Similarly, the success of norfloxacin-loaded collagen/chitosan sponges in full-thickness wound healing in rat models demonstrated effective wound healing with no adverse reactions, further emphasizing chitosan’s therapeutic versatility. Shimizu et al. (2014) further contributed by creating a hyaluronic acid (HA) spongy sheet loaded with arginine, a vitamin C derivative, and EGF. This innovative wound dressing demonstrated enhanced granulation tissue formation and angiogenesis in diabetic mice, underscoring the role of biochemical augmentation in promoting wound repair.

Maarof et al. (2019) developed an acellular dermal collagen graft enriched with fibroblast-conditioned medium. Tested in a mouse model, this collagen-based graft accelerated healing rates and achieved complete reepithelialization without signs of rejection, reinforcing its potential for use as a biomaterial in wound care.

To create a more sophisticated skin replacement, Climov et al. (2016) introduced an autologous skin construct (ASC) using porcine dermal fibroblasts and keratinocytes. This bilayered ASC model achieved robust integration and early vascularization, outperforming traditional cultured skin substitutes (CSS) with integration rates exceeding 90%, which typically integrate at rates of 71%–82% (Shores et al., 2007; Gosain et al., 2006). The ASC also minimized inflammation and wound contraction, underscoring its enhanced durability and stability, which are critical in addressing donor site limitations and risks tied to allogeneic grafts. Histological assessments highlighted ASC’s early angiogenesis, organized collagen bundling, and proper epidermal stratification, promoting stability and aesthetic outcomes comparable to native skin. Additionally, ASC-treated wounds exhibited limited contraction—a notable improvement over bilayered living cellular constructs (BLCC). BLCCs, such as Apligraf, consist of fibroblasts embedded in a bovine collagen matrix and a stratified keratinocyte layer. While effective for secondary-intention healing in chronic wounds, BLCCs are prone to sloughing and moderate contraction in full-thickness wounds due to limited cellular infiltration and vascular integration (Eaglstein et al., 1999; Falanga, 1998)—challenges ASC addresses through its self-assembled ECM, which fosters spontaneous vascularization and durable engraftment.

Building on collagen-based constructs, Strenge et al. evaluated the performance of silk fibroin in wound healing through a human 3D *ex vivo* model. Using both cast membranes and electrospun nonwoven matrices, they demonstrated improved early wound closure, rapid keratinocyte proliferation, and effective tissue integration. These findings highlight silk fibroin’s compatibility and potential in advanced wound care, particularly in applications requiring stable biocompatible materials. Arasteh et al. (2020) produced a bilayered skin substitute by electrospinning of silk fibroin on the human amniotic membrane, which had the ability to accelerate skin regeneration of full-thickness skin wounds in mice by reduction of inflammation, improvement of neovascularization, and limitation of scarring.

Miyazaki et al. (2019) developed a pre-vascularized 3D skin substitute to improve engraftment rates. Using a scaffold-free, layer-by-layer (LbL) cell coating technique with fibronectin and gelatin, this construct contained its own vascular network. In SCID mice models, the pre-vascularized grafts rapidly integrated with host vasculature within 7 days, reducing necrosis and tissue detachment compared to non-vascularized controls. These results indicate that pre-vascularized constructs offer stability, faster healing, and the potential to reduce immune rejection, suggesting future applications for complex wound repair. Further advancing vascularization, Won et al. (2019) utilized powdered, freeze-dried decellularized extracellular matrix (dECM) as bioink for 3D bioprinting. This approach preserved ECM proteins such as collagen and glycosaminoglycans, facilitating the creation of cellular dermal constructs with improved wound healing capabilities. This method offered a scalable, stable model for creating vascular-friendly grafts that align well with natural tissue integration processes.

In other vascularization strategies for treating full-thickness burns, studies highlight the critical role of stable vascular connections for effective wound healing (Frueh et al., 2017). The limited diffusion range of only 0.1–0.2 mm for oxygen and nutrients often complicates healing when grafting is delayed, commonly resulting in infections and scarring (Jain et al., 2022). Innovative approaches like pre-vascularized grafts have shown promise in preclinical models, reportedly enhancing the healing process by facilitating quicker integration with host tissues (Masson‐Meyers and Tayebi, 2021; Frueh et al., 2018).

The development of engineered skin constructs has expanded from basic wound coverage to include functional appendages and neuroregenerative elements, which are critical for restoring physiological functions such as thermoregulation and sensation. Li et al. (2015) focused on recreating essential skin appendages by embedding human sweat gland cells (SGCs) within a Matrigel™ matrix to reconstruct sweat glands in athymic nude mice. The resulting 3D structure formed sweat gland-like features and expressed key sweat-related proteins, demonstrating the potential to incorporate functional appendages into engineered skin. This advancement is particularly valuable for thermoregulation, as sweat glands play a crucial role in maintaining body temperature and skin homeostasis. The study underscores the promise of engineered skin grafts that go beyond structural repair to provide physiological functionality.

Restoring sensory functions in engineered skin grafts requires promoting neuroregeneration at the graft site, a challenge that has led researchers to explore stem cell-based solutions (Li et al., 2021; Lischer et al., 2023). Skin-derived precursor stem cells and induced pluripotent stem cells (iPSCs) have shown potential for nerve regeneration due to their ability to differentiate into neural lineages (Laurens et al., 2006). Wang et al. (2011) advanced this approach by developing tissue-engineered nerve conduits using iPSC-derived neural crest stem cells (NCSCs) seeded into nanofibrous tubular scaffolds. In a rat model with transected sciatic nerves, these NCSC-engrafted conduits promoted accelerated nerve regeneration, including axonal myelination, without teratoma formation for up to 1 year. Histological analysis confirmed that NCSCs differentiated into Schwann cells and integrated into the myelin sheath, highlighting the potential for iPSC-based constructs to provide sensory regeneration in skin graft applications.

Building on these findings, Blais et al. (2009) demonstrated nerve function recovery in a mouse model using collagen/chitosan sponges pre-seeded with murine Schwann cells, human skin fibroblasts, and keratinocytes. This scaffold achieved a current perception threshold similar to that of normal skin, underscoring the importance of Schwann cells for sensory restoration. The study illustrates how combining collagen-based scaffolds with cellular components can enhance neuroregenerative outcomes, an essential feature for restoring sensation in skin grafts.

In addition to functional and neuroregenerative properties, the stability and biocompatibility of engineered skin constructs are crucial for clinical applications. An innovative composite cultured skin (CCS) approach by combining a biodegradable polyurethane (PUR) scaffold with a Biodegradable Temporising Matrix (BTM) provides both dermal and epidermal support. Unlike traditional collagen scaffolds, PUR is fully synthetic, biodegradable, and associated with reduced inflammation, toxicity, and immune responses (Greenwood et al., 2020). The BTM-CCS method employs a two-stage process for wound closure, in which a 1 mm thick PUR scaffold, integrated with the patient’s fibroblasts within a fibrin network, is overlaid with a keratinocyte layer (Schlottmann et al., 2022). Early tests in porcine models demonstrated that CCS grafts were effective in wound healing, offering smooth, pliable skin and reducing the need for surgical intervention (Dearman et al., 2014; Dearman and Greenwood, 2021). This method represents a promising alternative for long-lasting, functional skin repair, overcoming limitations seen in conventional grafting materials.

### Clinical applications

The use of engineered skin substitutes in clinical settings has advanced significantly, with numerous studies evaluating their efficacy for wound repair, functional restoration, and long-term stability. Comparative analyses, case studies, and clinical trials demonstrate how these biomaterials support wound healing, reduce complications, and improve patient outcomes across various types of wounds and conditions. Kianian et al. (2023) conducted a detailed review and analysis to evaluate how well autologous skin grafts perform in comparison to engineered skin substitutes, including both acellular and cellular varieties, in terms of their ability to repair wounds (Kianian et al., 2023). Acellular constructs in the study included those derived from decellularized human or animal tissue or protein-based scaffolds, while cellular constructs involved matrices seeded with cells such as keratinocytes or fibroblasts. The meta-analysis included 66 studies involving 4,076 patients. There were no significant disparities observed in graft failure rates (*p* = 0.07) or the percentage difference in re-epithelialization (*p* = 0.92) when comparing the application of split-thickness skin grafts alone versus when co-grafted with acellular tissue-engineered constructs. Both groups exhibited similar mean scores on the Vancouver Scar Scale (*p* = 0.09). Overall, weighted averages from combined findings did not indicate any statistically significant variances in re-epithelialization or failure rates between epidermal cellular tissue constructs and split-thickness skin grafts (*p* = 0.55).

In treating acute full-thickness skin defects, Matsumine et al. (2019) applied an FGF-impregnated collagen-gelatin sponge, which facilitated rapid wound closure without complications. Similarly, commercially available options like Biobrane, a semi-synthetic bilayer wound dressing composed of a silicone outer layer and a nylon mesh coated with porcine collagen, are used for donor sites and partial-thickness burns. Biobrane provides a temporary covering that supports epithelialization and reduces fluid loss. When applied within 24 h post-injury, it demonstrated significant reductions in inpatient treatment durations, shortening hospital stays by 46% (*p* < 0.001) (Feng et al., 2016; Lesher et al., 2011). This efficacy highlights the potential of bioactive substitutes in enhancing wound healing efficiency and reducing the treatment burden.

In a unique application, Apligraf, a bilayered human skin substitute developed from neonatal foreskin, was successfully used in a newborn with the Dowling-Meara variant of epidermolysis bullosa (EB), a severe mechanobullous disorder. Unlike traditional supportive care focused on dressings and antibiotics, the Apligraf application led to complete healing of erosions within 3 days on treated areas, which remained resilient to trauma-induced blister formation. Untreated areas continued to develop lesions, highlighting the graft’s efficacy in providing rapid, durable wound closure and reducing sepsis risk in high-vulnerability cases. This outcome underscores the transformative potential of bioengineered skin in treating complex dermatological conditions like EB (Falabella et al., 1999).

Focusing on post-surgical wound care, Gohari et al. (2002) evaluated the use of Human Skin Substitute (HSS) for full-thickness wounds following Mohs or excisional surgery for skin cancer. HSS, which includes both allogenic and autologous substitutes, offers bioengineered skin materials to aid in wound healing. Allogenic HSS is derived from donor tissue and acts as a temporary covering, reducing infection risk and providing a favorable environment for healing until the patient’s skin can regenerate. In contrast, autologous HSS is created from the patient’s own cells, minimizing immune rejection risks and providing a more permanent graft that integrates well with existing tissue. Among 12 assessable patients, HSS promoted complete wound healing in 100% of cases over 6 months, showing re-epithelialization rates comparable to secondary intention healing. Importantly, HSS achieved superior cosmetic outcomes, resulting in more pliable, less vascular scars and enhanced patient satisfaction. This study highlights the role of HSS as a viable alternative for post-surgical wound management, especially for patients prioritizing aesthetic results.

Kolenik et al. (1999) studied the use of a lyophilized type I bovine collagen matrix (SkinTemp) in cases following Mohs surgery, particularly when immediate reconstruction was not feasible. The bovine collagen matrix expedited healing, reducing average healing times to 6.1 weeks compared to 9.4 weeks with traditional methods, with fewer dressing changes required per week. SkinTemp demonstrated a favorable safety profile with no infections or allergies reported, positioning it as a practical option to minimize wound care burdens and accelerate recovery.

A two-phase composite cultured skin (CCS) approach was applied to a patient with burns covering 95% of total body surface area (TBSA). CCS covered 40% of the initial burn area and provided durable, pliable skin with minimal intervention required for releasing skin tightness after 1.5 years. The CCS-treated areas showed scar outcomes comparable to traditional grafts and allowed smooth integration, with enhanced patient mobility and minimal cosmetic imperfections (Greenwood et al., 2020). This case demonstrates the potential of CCS in treating large-scale burns, offering robust long-term results with high patient satisfaction.

In a review of 130 patients, Bascone et al. (2023) examined the use of Integra bilayer wound matrix for facial reconstruction post-Mohs surgery. The bilayer matrix, effective in both single- and dual-stage reconstructions, achieved a 90.2% success rate, with 32-day re-epithelialization and 170-day re-pigmentation times. The low complication rate (12.8%) and reduced need for autologous tissue harvesting emphasize Integra’s advantages in aesthetic and functional restoration. Notably, dual-stage reconstruction correlated with increased aesthetic enhancement procedures, suggesting that Integra offers both functional repair and flexibility for aesthetic improvements. Regardless of material used, re-pigmentation rates vary among patients. There is a substantial delay for re-pigmentation in patients over 30 years old, with efficacy dropping below 65% (Mulekar et al., 2007).

Chalmers et al. (2010) further validated Integra^®^ in complex reconstructions following cancer excisions extending to bone or tendon. In 14 cases with digital and scalp wounds, Integra^®^ achieved an 87% graft take, with only minor complications in a subset of patients. This study supports the use of Integra^®^ for cases where traditional methods may not suffice, indicating its effectiveness in achieving durable healing even in challenging anatomical regions. Another multicenter trial assessed Integra^®^ Dermal Regeneration Template (IDRT) in treating chronic diabetic foot ulcers (DFUs). Patients receiving IDRT showed a 51% DFU closure rate at 16 weeks, outperforming standard care (32%, *p* = 0.001). This accelerated healing rate, coupled with improved quality of life, reinforces IDRT’s value in managing chronic wounds (Driver et al., 2015).

For auricular reconstruction, a synthetic nanofiber matrix was used by researchers in a case series of four patients with non-melanoma skin cancers. The matrix, applied to Mohs surgery wound beds, facilitated healing within 7.9 weeks on average and produced aesthetically favorable outcomes with minimal scarring and no deformities. This study points to the utility of nanofiber matrices in areas with complex anatomical structures, offering a promising alternative when secondary intention or full-thickness skin grafts (FTSGs) are not ideal (Zaiac et al., 2023).

Lembo et al. (2020) investigated Pelnac^®^, a new artificial dermis, in facial and scalp reconstructions. Over a follow-up period averaging 30 months, 93.75% of cases achieved complete graft intake with significant cosmetic improvement. The minimal complication rate and favorable Vancouver Scar Scale outcomes position Pelnac^®^ as an effective tool in reconstructive surgery, particularly for patients with prior surgical or radiation histories.

In a clinical phase I trial, Meuli et al. (2019) tested autologous dermo-epidermal skin substitutes in pediatric patients with deep partial- and full-thickness skin defects. The bioengineered grafts achieved stable integration with a 78% graft take by day 21 and skin closely resembling native tissue upon histological examination. This study illustrates the potential of patient-specific, cell-derived grafts for long-term regenerative outcomes, particularly for pediatric patients with limited donor sites.

Addressing the need for full-thickness wound repair, Mundinger et al. (2020) introduced an autologous homologous skin construct (AHSC), created from a small biopsy of the patient’s healthy skin. In a cohort of 15 patients, AHSC provided full-thickness skin regeneration with no adverse reactions and favorable structural and functional outcomes. This technique holds potential for complex wound repair, offering a sustainable alternative to extensive graft harvesting and minimizing donor-site morbidity.

In treating extensive burns, engineered skin substitutes have shown notable benefits. Boyce et al. (2017) compared autologous engineered skin substitutes (ESS) to traditional split-thickness autografts (AG) in 16 pediatric patients, with an average burn coverage of 76.9% total body surface area (TBSA). The study revealed that ESS required significantly less donor skin, with a closed wound area-to-donor skin area ratio of 108.7 for ESS versus 4.0 for AG. ESS achieved an engraftment rate of 83.5%, slightly lower than the 96.5% for AG, but offered substantial survival benefits; the mortality rate among ESS patients was 6.25%, significantly lower than the 30.3% observed in the National Burn Repository.

Another crucial finding was the development of antibodies to the biopolymer scaffold used in ESS, which was not significant in patient sera post-treatment. This indicates good biocompatibility of the ESS materials, which is essential for their acceptance and function as skin substitutes. Nevertheless, this approach comes with constraints, including the absence of additional cell types and adnexal structures, as well as the contraction of the collagen scaffold during the fabrication of ESS, alongside the relatively high cost and regulatory challenges. Furthermore, it is not currently available for commercial use. Recent preclinical investigations, however, have shown promising results by integrating melanocytes (Boyce et al., 1993; Duval et al., 2012), microvascular endothelial cells (Supp et al., 2002; Tremblay et al., 2005), mesenchymal stem cells (Bhowmick et al., 2016; Huang et al., 2012), sensory nerve cells (Blais et al., 2013), and hair follicle progenitor cells (Sriwiriyanont et al., 2013; Agabalyan et al., 2017) into the ESS framework. These findings illustrate ESS’s potential for large burns, though improvements are needed to enhance stability and long-term adherence.

Addressing specific clinical challenges, a study examined the clinical outcomes of 16 individuals who underwent treatment with ESS between 2007 and 2010 (Boyce et al., 2017). Notably, among patients with full-thickness burns encompassing more than 50% of their Total Body Surface Area (TBSA), ESS therapy showed a significant reduction in the need for harvesting donor skin grafts and a decrease in mortality rates compared to data from similar patient cohorts recorded in the American Burn Association’s National Burn Repository. The resulting closed wounds from ESS displayed both structural and functional similarities to natural skin. Nevertheless, this model encounters limitations, such as the absence of various cell types and adnexal structures, contraction of the collagen scaffold during ESS production, and complexities related to cost and regulatory hurdles, leading to its unavailability for commercial use.

Another promising burn treatment is TransCyte, an advanced dressing enriched with neonatal fibroblasts. These fibroblasts are cultured for 17 days, during which they produce essential components such as fibronectin, type I collagen, proteoglycan, and various growth factors. Despite the fibroblasts becoming non-metabolic post-freezing for storage, the clinical outcomes are noteworthy (Noordenbos et al., 1999). Lukish et al. (2001) examined its effectiveness in pediatric patients with partial-thickness burns. In this study, 92 patients treated with TransCyte showed reduced hospital stays compared to conventional therapy, highlighting the product’s ability to expedite healing and reduce the need for secondary skin grafts (Amani et al., 2006). TransCyte’s neonatal fibroblasts, cultured to produce essential extracellular matrix (ECM) components, demonstrated benefits in terms of healing efficiency and clinical outcomes, particularly in pediatric burns where treatment options are constrained.

Patients suffering from extensive burns often experience the loss of hair and sweat glands. While primarily cosmetic, the absence of sweat glands can affect thermoregulation, making the reconstruction of sweat glands important (Anyanwu and Cindass, 2024). Still, most commercially available skin substitutes fail to provide both the epidermal and dermal layers necessary for effective treatment of burn wounds. For example, Epicel, a cultured epidermal autograft, reconstructs only the epidermis and does not address the dermal layer. As a result, treatments like Epicel often leave the regenerated skin lacking elasticity and mechanical stability (Wang et al., 2006). This highlights the critical need for skin substitutes that integrate both epidermal and dermal components. Research indicates that the restoration of dermal connective tissue significantly aids the healing of excised full-thickness burns. The fibrovascular connective tissue not only enhances the mechanical strength of the epidermis but also supplies the necessary blood flow for nourishment during healing (Boyce, 1998).

Despite current shortcomings for skin grafting in burn wounds, tissue-engineered skin presents a promising solution. Unlike conventional substitutes, it includes autologous fibroblasts and keratinocytes cultured on a scaffold, utilizing cells derived from a split-thickness skin biopsy from the patient. This engineered skin effectively heals full-thickness burn wounds by providing both the crucial epidermal and dermal components, enabling functional wound closure (Supp and Boyce, 2005). Clinical evidence further supports that this approach can permanently replace both skin layers in a single grafting procedure (Boyce and Warden, 2002; Boyce et al., 1995; Boyce et al., 1999). Additionally, tissue-engineered skin has shown effectiveness in treating extensive burns covering more than 50% of TBSA (Boyce and Warden, 2002; Boyce et al., 1999) and in managing large congenital nevi (Passaretti et al., 2004), demonstrating its broad potential in advanced skin repair.

## Future directions and overcoming limitations

Despite the promising advancements in BSGs, several key limitations impede their widespread clinical adoption. Firstly, ensuring biocompatibility and minimizing immune reactions present significant challenges, as allogeneic and xenogeneic components can provoke immune responses that lead to graft rejection or failure (Petrus-Reurer et al., 2021). Nonetheless, the use of xenogeneic-derived biologicals such as bovine, rat, or porcine collagens or glycosaminoglycans in creating a dermal-epidermal equivalent poses risks such as immune rejection and the potential transmission of prions (Shahrokhi et al., 2014; Gallo et al., 2020). Even human-derived biologics are not without risks, but these are generally lower than those associated with xenogeneic materials due to differences in biocompatibility (Fishman, 2018). Additional hurdles are achieving integration with host tissues and establishing a functional vascular network within the graft, which are crucial for long-term success but often remain unmet by current technologies (Phua et al., 2021). Furthermore, the production of BSGs involves complex and expensive biotechnological processes, posing difficulties in scaling these treatments to be affordable and accessible while maintaining consistent graft quality (Metcalfe and Ferguson, 2007).

While BSGs may involve higher upfront expenses due to their advanced bioengineering requirements, studies suggest they can help reduce total treatment costs by decreasing the need for repeated surgeries, lengthy hospital stays, and extended recovery times for severe cases (Langer and Rogowski, 2009). In comparison, ASGs are generally more affordable for smaller wounds, though their cumulative treatment costs can rise with additional procedures. Addressing these cost and access differences will be important to ensure equitable treatment across healthcare settings, particularly where resources are constrained (Elkady et al., 2024).

Aesthetically and functionally matching the patient’s original skin also poses a challenge, as issues such as color mismatch, texture differences, and inadequate mechanical properties can affect patient satisfaction (Shin et al., 2021). As Dearman et al. (Lin et al., 2022) pointed out, employing a synthetic scaffold and autologous cell approach could mitigate these concerns. In addition to this, we suggest that utilizing decellularized ECM from human tissues as a scaffold for skin regeneration could provide an environment closely resembling native dermal architecture. This approach encourages the integration and growth of patient-derived cells while minimizing the risk of immune rejection. Lastly, there is a need for long-term studies to understand the durability, risk of complications, and overall impact of BSGs on patient health and recovery (Dearman et al., 2021).

To minimize immune reactions and enhance biocompatibility, targeted genetic modifications of donor cells to downregulate MHC molecules or to express immune-evasive molecules such as PD-L1 could be a particularly useful strategy. The development of scaffolds that can locally release immunosuppressive cytokines or use encapsulated immunomodulatory agents like corticosteroids or tacrolimus could help in reducing graft rejection rates. Incorporating hydrogels with bioactive agents that mimic the anti-inflammatory environment might also prevent immune-mediated graft destruction (Zakrzewski et al., 2014). Genetic engineering strategies could involve CRISPR-Cas9 mediated knock-in/knock-out techniques to introduce immunomodulatory genes into the donor cells’ DNA, such as those coding for immune checkpoint molecules like PD-L1 (Allemailem et al., 2023). Scaffolds could be biofunctionalized with nanoparticle-based delivery systems that release corticosteroids or tacrolimus in a controlled manner. Such a system could use pH-sensitive liposomes or polymer-based microspheres that respond to the inflammatory microenvironment, ensuring targeted delivery (Zhuo et al., 2020). Further, hydrogels could be synthesized to release soluble factors like TGF-β or IL-10, exploiting the natural anti-inflammatory pathways to create a conducive environment for graft acceptance (Kafili et al., 2024).

Additionally, achieving robust integration and vascularization remains a significant hurdle. Advanced bioprinting techniques can now incorporate synthetic microvasculature designed to match the hierarchical branching patterns of the host’s vascular network. Using computational modeling to predict the optimal architectural layout, these structures would be bioprinted with bioinks composed of endothelial progenitor cells and pericytes (Tripathi et al., 2023; Ng et al., 1970). Scaffold materials may include angiogenic factor-conjugated bioactive peptides or ECM components like hyaluronic acid that are cross-linked to create a matrix with controlled degradation rates, thereby releasing VEGF or FGF in a sustained manner (Shokrani et al., 2022). Mechanotransducive properties of scaffolds could be tuned using biomaterials with graded stiffness to guide cellular behavior, especially fibroblasts’ myofibroblast differentiation, which is pivotal in wound contraction and matrix deposition (Wang F. et al., 2023).

Creating a multi-layered cellular structure involves culturing melanocytes, fibroblasts, and keratinocytes in a tiered bioreactor system that allows sequential deposition of cells, each within their own micro-environmental niche. Melanocytes could be genetically modified to optimize melanin production, matching the patient’s skin tone. Fibroblasts could be sourced from the patient’s adipose tissue, ensuring a robust source of autologous cells for ECM production. Dermal fibroblasts could be pre-treated with mechanotransducive cues to prime ECM synthesis pathways (Lee et al., 2009). Integration of bioactive cues such as elastin-like polypeptides could enhance the elastic properties of the dermal component. For the epidermal layer, keratinocyte stem cells could be sourced and expanded in a medium supplemented with epidermal growth factors to enhance stratification and differentiation (Sarangthem et al., 2021).

BSG manufacturing processes can be automated by implementing robotic arms equipped with sensors to monitor cell growth and scaffold deposition in real time, adjusting parameters dynamically for optimized production (Mathew et al., 2022; Doulgkeroglou et al., 2020). Continuous flow bioreactors can be designed with biodegradable microcarrier systems to enhance cell proliferation and ease of harvest (Tsai and Pacak, 2021). 3D bioprinters should be integrated with machine learning algorithms that adapt printing parameters in response to real-time tissue growth feedback, ensuring precision in scaffold structure and cellular composition (Ning et al., 2023). Furthermore, modular graft systems would benefit from “smart” biomaterials that respond to wound exudates and adapt their mechanical and degradation properties accordingly, providing a personalized approach to wound healing. Additionally, modular graft systems that allow customization for individual wounds could optimize resource use and minimize waste, thereby making the process more cost-effective (Liu et al., 2023; Augustine et al., 2014).

Artificial intelligence (AI) holds potential in the field of bioengineering, particularly in the development and optimization of skin substitutes (Du‐Harpur et al., 2020). One of the primary ways AI can contribute is through the enhancement of bioprinting technologies. By integrating AI with 3D bioprinting, algorithms can optimize the placement of cells and biomaterials, adapting in real-time to the specific wound topography and patient’s skin characteristics. This ensures that the structural and functional aspects of the skin, such as thickness, elasticity, and barrier properties, are precisely tailored to individual needs (Freeman et al., 2022). Furthermore, AI can play a critical role in the design and development of biomaterials used in skin substitutes. Through machine learning models that predict the behavior of biomaterials under various conditions, researchers can rapidly identify the most effective combinations of materials and cellular components. This accelerates the iterative process of testing and refining skin substitutes, reducing both time and cost (Negut and Bita, 2023). In cellular therapy, AI can enhance stem cell technologies by predicting the differentiation pathways of stem cells into desired cell types, such as keratinocytes or dermal fibroblasts (Srinivasan et al., 2021). By analyzing vast datasets from previous experiments, AI can identify patterns and factors that influence cell behavior, guiding the development of protocols that yield more efficient and stable results (Nosrati and Nosrati, 2023). AI also extends its utility to the regulatory and clinical trial phases. This not only helps in fine-tuning the clinical protocols but also in personalizing treatment approaches, ensuring higher success rates and better patient outcomes (Vora et al., 2023). Additionally, AI can monitor and analyze the performance of skin substitutes post-implementation, using data from follow-up visits to predict long-term outcomes and potential complications. This ongoing evaluation can inform future improvements in skin substitute formulations and application techniques (Bohr and Memarzadeh, 2020).

## Conclusion

In this review, we assessed the efficacy of BSGs and ASGs for treating various skin defects. Drawing from a comprehensive selection of *in vitro*, *in vivo*, pre-clinical, and clinical studies, we evaluated these grafting methods in terms of wound healing, tissue integration, immunogenicity, and functional outcomes—key factors in the clinical approach to skin reconstruction.

ASGs demonstrated superior initial healing rates due to their immediate integration and immune compatibility, making them especially effective in scenarios demanding rapid wound closure, such as acute burns and extensive surgical wounds (Kenny et al., 2024; Šuca et al., 2024; Maskan Bermudez et al., 2024). BSGs, in contrast, achieved comparable long-term healing results and are particularly beneficial when donor skin availability is limited or to minimize donor site morbidity. Significant advances in BSG scaffold technology and cellular integration—such as the addition of growth factors and cytokines—have enhanced their healing efficacy, allowing them to match ASGs over time (Phua et al., 2021; Boyce and Lalley, 2018; Aleman Paredes et al., 2024). Pre-vascularized BSGs, for instance, promoted quicker, more durable integration within host tissue (Chen et al., 2017).

Immunogenicity differed between graft types. ASGs, by nature, exhibited lower immunogenicity, while BSGs’ immune response varied depending on their cellular composition (Dixit et al., 2017). Acellular BSGs generally trigger fewer immune reactions, whereas cellular constructs, especially those using allogeneic or xenogeneic cells, require more careful immunogenicity management to mitigate rejection risks (Chen et al., 2017; Mohammadyari et al., 2023).

Both ASGs and BSGs demonstrated strong functional outcomes. ASGs provided durability and structural integrity, essential for high-aesthetic areas, such as facial reconstructions (Kamolz et al., 2022; Zan et al., 2024). BSGs, however, have proven beneficial for chronic wounds and non-healing ulcers, offering improved outcomes where traditional ASGs might not be feasible. Aesthetic outcomes and patient satisfaction were high for both graft types, though ASGs were generally preferred for regions requiring superior cosmetic results (Qin et al., 2022; Hahn et al., 2022). Meanwhile, BSGs have advanced significantly, with innovations that enable better matching of natural skin’s color and texture (Boyce et al., 1993; Boyce and Warden, 2002; Boyce and Lalley, 2018).

BSGs are particularly valuable for treating large skin deficits, where the limitations of ASGs—such as limited donor tissue and donor site morbidity—make ASGs impractical. While STSGs used in large wound coverage often yield suboptimal cosmetic results, autologous engineered skin offers immediate coverage with a stratified epidermis, reducing scarring, pain, and itch, and minimizing donor skin morbidity (Dearman et al., 2021). Bilayered graft approaches present unique strengths and limitations, suggesting that the ideal solution may combine biopolymer scaffolds with stem cells to create functional, clinically effective alternatives (Zhang et al., 2020).

BSGs offer a promising pathway forward. Designed to address traditional grafting limitations, they incorporate biocompatible materials, growth factors, cytokines, and cellular elements to enhance tissue integration and healing. However, BSGs still face challenges in achieving robust tissue integration, managing immune responses, and replicating natural skin’s functionality (Dixit et al., 2017). These issues highlight the need for further research in tissue engineering and regenerative medicine. Ongoing research into BSGs should focus on enhancing graft material compatibility, reducing immunogenicity, and improving integration with the host tissue. Developing pre-vascularized bioengineered grafts with the capacity for rapid integration with the patient’s circulatory system could transform treatment for large or complex wounds (Urciuolo et al., 2019). Research efforts should also aim to create scalable, cost-effective BSG production methods, improving accessibility and consistency in therapeutic efficacy.

Our findings underscore a shift in clinical practice, advocating a selective application of BSGs while recognizing the sustained value of ASGs. Continued innovation and clinical trials are essential to improve BSG biocompatibility, integration, and immune profiles. By optimizing both graft types, future strategies can enhance patient outcomes across diverse reconstructive needs. Further research will drive these advancements, fostering more personalized, effective skin reconstruction techniques.

## Data Availability

The original contributions presented in the study are included in the article/Supplementary Material, further inquiries can be directed to the corresponding author.
