# Supplementary material for: Advancements in bioengineered and autologous skin grafting techniques for skin reconstruction: a comprehensive review
Source: Front Bioeng Biotechnol. 2025 Jan 7;12:1461328. doi: 10.3389/fbioe.2024.1461328 (PMC11747595; doi:10.3389/fbioe.2024.1461328)
Supplement: Supplementary file 1 [file Supplementaryfile1.docx]

**Abbreviations**

Acellular dermal matrix (ADM)
Adipose-derived stem cell (ADSC)
Antigen-presenting cell (APC)
Arteriovenous loop (AV-loop)
Autologous Homologous Skin Construct (AHSC)
Autologous skin construct (ASC)
Autologous skin graft (ASG)
Bilayered living cellular construct (BLCC)
Bioengineered skin graft (BSG)
Biodegradable Temporising Matrix (BTM)
Chitosan dermal substitutes (CDS)
Composite cultured skin (CCS)
Composite skin substitute (CSS)
Cultured epithelial substitute (CES)
Decellularized extracellular matrix (dECM)
Diabetic foot ulcer (DFU)
Endothelial growth factor (EGF)
Engineered skin substitutes (ESS)
Epidermolysis bullosa (EB)
Epithelial stem cells (EpSC)
Extracellular matrix (ECM)
Fibroblast growth factor (FGF)
Full-thickness skin graft (FTSG)
Glycosaminoglycans (GAGs)
Growth factors (GFs)
Hepatocyte growth factor (HGF)
Human Skin Substitute (HSS)
Hyaluronic acid (HA)
Induced pluripotent stem cell (iPSC)
Integra® Dermal Regeneration Template (IDRT)
Interferon-gamma (IFN-γ)
Interleukin (IL)
Interleukin-1 (IL-1)
Interleukin-6 (IL-6)
Layer-by-layer (LbL)
Major histocompatibility complex (MHC)
Matrix metalloproteinase (MMP)
Mesenchymal stem cell (MSC)
Mitogen-activated protein kinase (MAPK)
Non-melanoma skin cancer (NMSC)
Nuclear factor-kappa B (NF-κB)
Platelet-derived growth factor (PDGF)
Polyurethane (PUR)
Programmed death-ligand 1 (PD-L1)
Randomized controlled trial (RCT)
Rat vascular endothelial cell (RVEC)
Scar outcome scale (SOSS)
Split-thickness autograft (AG)
Split-thickness skin graft (STSG)
Stromal vascular fraction (SVF)
Sweat gland cell (SGC)
Total body surface area (TBSA)
Transforming growth factor-beta (TGF-β)
Tumor necrosis factor-alpha (TNF-α)
Vascular endothelial growth factor (VEGF)
Vascular endothelial growth factor receptor (VEGFR)
Vascular smooth muscle cell (VSMC)
Venous leg ulcers (VLUs)
